# Patient-reported perioperative anaesthesia-related anxiety is associated with impaired patient satisfaction: a secondary analysis from a prospective observational study in Switzerland

**DOI:** 10.1038/s41598-023-43447-6

**Published:** 2023-09-28

**Authors:** Corina Bello, Matthias Nuebling, Kira-Lee Koster, Thomas Heidegger

**Affiliations:** 1 Department of Anaesthesiology Spital Grabs, Spitalregion Rheintal Werdenberg Sarganserland, Grabs, Switzerland; 2 Department of Anaesthesiology and Pain Medicine, Bern University Hospital, University of Bern, Bern, Switzerland; 3 Data and Consulting, GDB mbH, Denzlingen, Germany; 4 Department of Medical Oncology and Haematology, Cantonal Hospital St. Gallen, St. Gallen, Switzerland

**Keywords:** Epidemiology, Outcomes research, Clinical trials

## Abstract

Perioperative anxiety is common. The relationship between anxiety and patient satisfaction with anaesthesia is still under debate. We assessed the prevalence and different causes of anaesthesia-related fears leading to perioperative anxiety and its association with patient satisfaction. A multiple-time validated, psychometrically developed questionnaire assessing the presence of anxiety, causes of fear, and different dimensions of patient satisfaction was sent to patients after discharge. The clinical data were obtained from a previous study. The sample size was calculated to recruit a minimum of 300 completed questionnaires. Statistical analyses included multivariate logistic regression models. Complete data were available for 474 of the 600 patients recruited for the study (response rate: 79%). A total of 141 patients (30%) reported anxiety regarding anaesthesia before hospital admission. The prevalence of anxiety was significantly associated with patient age (< 54 years: n = 196, prevalence = 37%; > 54 years: n = 263, prevalence = 24%; p = 0.002), female sex (female: n = 242, prevalence 39%; male: n = 223, prevalence 20%; p < 0.001), and surgical speciality (gynaecology (n = 61, prevalence = 49%), otolaryngology (n = 56, prevalence = 46%); p < 0.001). Fear of not awakening from anaesthesia (n = 44, prevalence = 32%, SD 45.8) and developing postoperative nausea or vomiting (n = 42, prevalence = 30%, SD 46.0) were the most reported anaesthesia-related causes of fear. Anxiety was associated with impaired overall patient satisfaction (mean dissatisfaction score 15%, versus 23%, SD 16.3 in the anxious group, SD 16.3, p < 0.001), especially regarding the dimensions “information and involvement in decision-making” (14% of deficits stated in the non-anxious group compared to 23% in the anxious group, p < 0.001), “respect and trust” (2% vs 6.26%, p < 0.001) and “continuity of care” (50% vs 57%, p < 0.015). Patient-reported anaesthesia-related anxiety is common and may affect important outcome parameters such as patient satisfaction. Abstract presented in e-poster format at Euroanaesthesia 2023, June 3–5, Glasgow.

## Introduction

Perioperative anaesthesia-related anxiety is an important concern in patients undergoing anaesthesia. Anxiety has been previously reported as the worst part of the perioperative period^1^. Pharmacological and non-pharmacological approaches to easing anxiety have been extensively studied. Nonetheless, patient-reported peri-operative anxiety remains common^2^. While there are many validated questionnaires to identify and assess the presence of anxiety^3^, most of them are not specific for detecting anaesthesia-related anxiety at the preoperative visit and none of them detect specific anaesthesia-related causes of fear. This is a prerequisite for the effective mitigation of anxiety.

In addition, anaesthesia-related anxiety may affect patient satisfaction, which is an important patient-reported outcome^4^. However, the relationship between preoperative anxiety and patient satisfaction remains unclear.

This secondary analysis of data from a previous prospective study had two main aims: to determine the prevalence of perioperative anaesthesia-related anxiety and the prevalence of specific causes of anaesthesia-related fears and to evaluate the potential association between perioperative anxiety and patient satisfaction using a psychometrically-developed, multiple-times validated questionnaire^5,6^.

## Methods

This study was based on a secondary analysis of data from a prospective cohort study on patient satisfaction with divided anaesthesia care. This study was approved by the Medical Ethics Committee of St. Gallen, Switzerland (Business Administration System for Ethics Committees 2017–00,090; Registry of all Projects in Switzerland—RAPS—registration number: 2017-00090)^7^. The requirement for informed consent for secondary analysis was waived by the Medical Ethics Committee of St. Gallen, Switzerland. We followed the Strengthening the Reporting of Observational Studies in Epidemiology (STROBE) reporting guidelines^8^. All participants received treatment according to the standards of the study centre. The study complied with the ethical principles of the Declaration of Helsinki.

All patients aged at least 16 years with American Society of Anesthesiologists (ASA) classification I-III from our anaesthesia consultation list were assessed for inclusion between October 2019 and February 2020. The inclusion criteria were elective inpatient surgery for general, orthopaedic, gynaecologic, otolaryngologic, maxillofacial, urologic, or plastic surgery. Patients who needed an emergency intervention, had insufficient German language reading comprehension ability, or had cognitive limitations were excluded.

Concomitant with the standard preoperative pathway at our institution, all patients were provided with a stepwise description of anaesthesia care in the form of an informative brochure, followed by a face-to-face inpatient visit for preoperative assessment and consent to the anaesthesia plan agreed upon during the visit. The preoperative assessment was led by resident and consultant anaesthetists. Patients were requested to complete a patient satisfaction questionnaire two weeks after discharge. The questionnaire, including a prepaid return envelope, was sent to patients’ homes after discharge to reduce social desirability bias (more positive assessments of the caregivers while still “under care” in fear of negative consequences)^9^.

The 55-item patient satisfaction questionnaire used in our study was psychometrically developed according to a stepwise protocol and retested several times for construct validity and reliability^5,7,10,11^. Three non-quality-of-care-related questions within the questionnaire specifically addressed anaesthesia-related anxiety (Table 1). The first question was regarding anxiety in general: “Did you feel anxious regarding the upcoming anaesthesia before hospital admission?”. If the response was “yes”, a list of nine subsequent specific causes of fear was presented as multiple-response questions. These causes included fear of not awakening from anaesthesia, fear of waking up during surgery, fear of being at mercy, and fear of developing postoperative nausea and vomiting (PONV)^4^. Finally, the third question was regarding the consultation of four potential groups of contact persons during the perioperative period for verbalisation of potential patient fears: anaesthetist, general practitioner, other physicians, friends and family, or no contact person^1^.

**Table 1:** Anxiety-related questions of the questionnaire.

Q1:	Did you feel anxious regarding the upcoming anaesthesia before hospital admission?
Q2:	What was the most important cause of your fear?
	(a) Fear of not awakening from anaesthesia
	(b) Fear of waking up during anaesthesia
	(c) Fear of being at mercy
	(d) Fear of postoperative nausea and vomiting
	(e) Fear of paralysis and damage of the spinal cord
	(f) Fear of problems with concentration, memory or forgetfulness
	(g) Fear of cardiovascular problems
	(h) Fear of pain
	(i) Other cause
Q3:	Were there any contact persons to talk about your fears during the perioperative period?
	(a) Anaesthetist
	(b) General practitioner
	(c) Other physicians
	(d) Friends and family
	(e) No contact person

The quality of care was assessed using 29 questions in the questionnaire. They investigated a broad range of potential quality-related “problems” giving a “total problem score” or “total dissatisfaction score” and sub-scores for deficits experienced for the following six dimensions: “information and involvement in decision-making”, “respect and trust”, “delays”, “nursing care in recovery room,” “continuity of personal care by anaesthetist.” and “pain management” (Supplement Koster)^7^. All problem scores ranged from 0% (no dissatisfaction with the related items) to 100% (maximal dissatisfaction with all quality items). The “total dissatisfaction score” was created using the number of problems reported in all 29 quality questions in the same manner, ranging between 0% (no dissatisfaction with all 29 quality items) and 100% (dissatisfaction with all 29 quality items).

We collected baseline and demographic data to ensure that no known or possibly unknown confounders affecting the different subdimensions or overall patient satisfaction were missed. Therefore, we aimed to minimise bias in the planned comparison of patient satisfaction between the anxious and non-anxious groups. Data on ASA classification^12^, anaesthetist performing the preoperative assessment (consultant, resident anaesthetist), anaesthetist in charge of anaesthesia (consultant, resident anaesthetist), BMI, days from preoperative assessment to surgery, length of hospital stay, hospital stays in the previous six months, surgical complications (Clavien-Dindo^13^), type of anaesthesia (general or regional anaesthesia, or monitored anaesthesia care (MAC)), surgical specialty (general, orthopaedic, gynaecologic, otolaryngologic, maxillofacial, urologic, or plastic surgery), and extent of surgery (minor, moderate, major) were collected from the hospital data records for all patients.

Data on age, sex, education level (primary, secondary, or tertiary), type of insurance (standard, semi-private, or private), and self-rated health (excellent, fair, or poor) were obtained from the questionnaire.

We originally aimed to recruit a minimum of 300 study participants with complete data for the analysis. With 300 samples, the 95% confidence interval (CI) (two-sided) for a proportion value of 80% (commonly used for satisfaction items) would be restricted roughly within ± 5% (75.1–84.1%)^14^.

Data were analysed using SPSS version 25 (SPSS Inc., Chicago, IL). Descriptive statistics included range, mean, and standard deviation (SD) for continuous data and numbers and percentages for discrete variables. Associations of patient-reported anaesthesia-related anxiety with demographic factors, dissatisfaction scores from the questionnaire, or data from the patient file were studied using analysis of variance (ANOVA) and multiple comparison of means (including the Scheffé test) or correlation analyses. Multivariate logistic regression analysis was conducted to build parsimonious multivariate models by simultaneously including significant bivariate predictive factors. For all analyses, p < 0.05 (two-tailed) was regarded as the threshold for significance.

## Results

**Figure 1:**
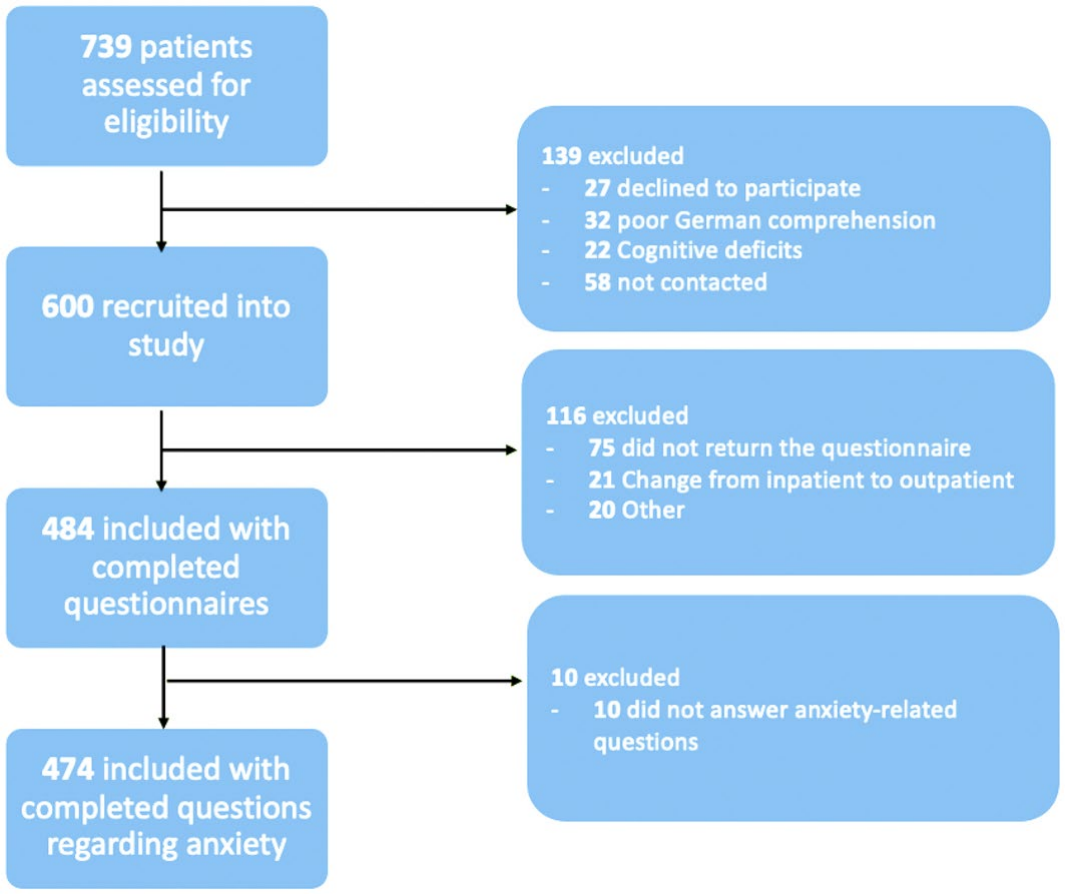
Patient flowchart.

Of the 739 patients available during the study period, 600 were recruited. Of these, 484 returned the questionnaire, and 474 (79% of the 600 enrolled) answered questions concerning anxiety. A patient flow diagram is shown in Fig. 1.

A total of 101 patients (21% of the 474) received regional anaesthesia, 370 (78%) received general anaesthesia, and only three (< 1%) received MAC. Sex was equally represented (51% female), and the mean patient age was 54.9 years (Table 2).

**Table 2:** Hospital data with baseline patient characteristics between patients with completed questionnaire regarding anxiety-related questions and recruited patients.

	Subcategories	Patients (n = 474)
Age, two groups	< 54 years	196 (41)
	≥ 54 years	263 (56)
Sex	Female	242 (51)
	Male	223 (47)
BMI	< 20	22 (5)
	20 to < 25	152 (32)
	25 to < 30	168 (35)
	30 to < 35	95 (20)
	≥ 35	37 (8)
Number of hospital admissions in last 6 months	none	408 (86)
	1	57 (12)
	2	8 (2)
	3	1 (0)
ASA Classification	I	134 (28)
	II	306 (65)
	III	34 (7)
Amount of time between preoperative evaluation and surgery	1 day	83 (18)
	1 day – 1 week	169 (36)
	> 1 week	222 (47)
Preoperative evaluation	Resident	219 (46)
	Consultant	255 (54)
Performance of anaesthesia	Resident	134 (28)
	Consultant	340 (71)
Type of anaesthesia	General anaesthesia	370 (78)
	Regional anaesthesia	101 (21)
	Monitored anaesthesia care	3 (1)
Extent of surgery	Minor	50 (11)
	Moderate	259 (55)
	Major	165 (35)
Surgical Specialty	General surgery	114 (24)
	Orthopaedics	188 (40)
	Gynaecology	61 (13)
	Otolaryngology	56 (12)
	Maxillofacial surgery	7 (1)
	Plastic surgery	6 (1)
	Urology	42 (9)
Complications (Clavien dindo classification)	< 3	467 (99)
	≥ 3	7 (2)
Health Status	Excellent	279 (59)
	Fair	151 (32)
	Bad	36 (8)
Insurance Status	Standard	295 (62)
	Semi-Private	104 (22)
	Private	67 (14)
Length of hospital stay	1–3 days	302 (64)
	4–6 days	127 (27)
	1–3 weeks	31 (7)
	> 3 weeks	3 (1)
Level of Education	Primary	83 (18)
	Secondary	224 (47)
	Tertiary	146 (31)

Values are absolute numbers and relative numbers (%, rest to 100% is missing answers).

Patient age was split into two groups based on mean-age.

A total of 141 patients (30%) reported anxiety regarding the upcoming anaesthesia. Among these patients, fear of not awakening from anaesthesia (n = 44, 32%) or developing PONV (n = 42, 30%) were the most reported causes of anxiety (Table 3).

**Table 3:** Prevalence of anaesthesia-related anxiety and specific causes of fear.

Q1: Did you feel anxious regarding the upcoming anaesthesia before hospital admission?	141 (29.7)
Q2: What is the most important cause of your fear?	
(a) Fear of not awakening from anaesthesia	44 (31.7)
(b) Fear of waking up during anaesthesia	18 (13.0)
(c) Fear of being at mercy	23 (16.6)
(d) Fear of postoperative nausea and vomiting	42 (30)
(e) Fear of paralysis and damage of the spinal cord	20 (14.4)
(f) Fear of problems with concentration, memory or forgetfulness	18 (13.0)
(g) Fear of cardiovascular problems	14 (10.1)
(h) Fear of pain	20 (14.4)
(i) Other cause	9 (6.5)
Q3: Were there any contact persons to talk about your fears during the perioperative period?	
(a) Anaesthetist	92 (66.2)
(b) General practitioner	17 (12.2)
(c) Other physicians	8 (5.8)
(d) Friends and family	74 (53.2)
(e) No contact person	6 (4.3)

Values are absolute numbers and relative numbers (%).

Of the patients who reported having anxiety, 92 (66%) were able to speak about their feelings with an anaesthetist. Only six patients (4%) reported not having anyone to speak about their fears (Table 3).

In the bivariate analysis, age, sex, and surgical speciality were significantly associated with the prevalence of anxiety in general; patients under 54 years of age reported significantly more anxiety (37%) than older patients (24%). Women had much higher anxiety prevalence values than men (39% vs. 20%, respectively). Moreover, patients undergoing gynecologic (49%) and otolaryngologic surgery (46%) had the highest anxiety rates compared to those undergoing general (26%), urologic (26), plastic (17%), and maxillofacial (14%) surgery. None of the other factors tested were significantly related to the prevalence of anxiety (Table 4).

**Table 4:** Bivariate analysis of anxiety.

	Subcategories	Q1. Anxiety (prevalenve in %)	Chi^2^-test for parameter: Coefficient Phi or Cramer’s V (signifinance)
Age, two groups	< 54 years	37.2	− 0.144 (0.002)
	≥ 54 years	24.0	
Sex	Female	39.3	− 0.213 (< 0.001)
	Male	19.7	
BMI	< 20	36.4	0.082 (0.532)
	20- < 25	32.9	
	25- < 30	29.8	
	30- < 35	23.2	
	≥ 35	29.7	
Number of hospital admissions in last 6 months	none	29.2	0.067 (0.542)
	1	31.6	
	2	50.0	
	3	0	
ASA Classification	I	28.4	0.030 (0.804)
	II	30.7	
	III	26.5	
Amount of time between preoperative evaluation and surgery	1 day	39.8	0.112 (0.052)
	1 day – 1 week	24.9	
	> 1 week	29.7	
Preoperative evaluation	Resident	30.6	0.020 (0.665)
	Consultant	28.8	
Performance of anaesthesia	Resident	28.2	0.053 (0.252)
	Consultant	33.6	
Type of anaesthesia	General anaesthesia	27.7	0.058 (0.454)
	Regional anaesthesia	30.5	
	Monitored anaesthesia care	0	
Extent of surgery	Minor	18.0	0.105 (0.074)
	Moderate	29.0	
	Major	34.6	
Surgical Specialty	General surgery	26.3	0.232 (< 0.001)
	Orthopaedics	22.3	
	Gynaecology	49.2	
	Otolaryngology	46.4	
	Maxillofacial surgery	14.3	
	Plastic surgery	16.7	
	Urology	26.2	
Complications (Clavien Dindo Classification)	< 3	29.6	0.035 (0.445)
	≥ 3	42.9	
Health status	Excellent	29.4	0.012 (0.969)
	Fair	30.5	
	Bad	30.6	
Insurance Status	Standard	32.9	0.113 (0.052)
	Semi-Private	28.9	
	Private	17.9	
Length of hospital stay	1–3 days	29.5	0.054 (0.720)
	4–6 days	28.4	
	1–3 weeks	38.7	
	> 3 weeks	33.3	
Level of education	Primary	28.9	0.027 (0.850)
	Secondary	31.7	
	Tertiary	30.5	

Chi^2^-tests. For 2*2 tables (df = 1) coefficient Phi is given is last column, for tables above 2*2 (df >  = 2) Cramer’s V is given.

Values are prevalence rates (%).

Omitting or collapsing small categories leads to almost unchanged results.

(a) Collapsing values 2 and 3 for “Number of hospital admissions in last 6 months” results in: Cramer’s V = 0.048 (0.581).

(b) Omitting Monitored anaesthesia care from “Type of anaesthesia” results in: Phi = 0.025 (0.584).

(c) Omitting Maxillofacial Surgery and Plastic surgery from “Surgical Specialta” results in: Cramer’s V = 0.229 (< 0.001).

(d) Collapsing groups 1–3 weeks and > 3 weeks for “Length of hospital stay” results in: Cramer’s V = 0.053 (0.522).

In a multivariate logistic regression model on the prevalence of anxiety testing, all parameters reached a bivariate significance level of p < 0.10 simultaneously (backward stepwise procedure, PIN 0.05, POUT 0.10), and age, sex, and surgical speciality remained significant independent predictors, confirming the bivariate relationship at a multivariate level (Model Ch^2^ = 47.0, df = 8, p < 0001, Nagelkerkes R^2^ = 0.13) (Table 5).

**Table 5:** Logistic Regression.

Variable	beta	SE (beta)	df	p	OR
Age (in years)	− 0.018	0.007	1	0.008	0.982
Sex	0.879	0.247	1	< 0.001	2.408
Surgical specialty			6	0.060	
Orthopaedics	− 0.162	0.292	1	0.580	0.8510
Gynaecology	0.500	0.360	1	0.165	1.648
Otolaryngology	0.667	0.368	1	0.070	1.948
Maxillofacial surgery	− 1.141	1.139	1	0.316	0.319
Plastic surgery	− 0.962	1.140	1	0.399	0.382
Urology	0.570	0.445	1	0.200	1.768
Constant	0.508	0.692		0.464	1.661

Sex coded as: male = 0, female = 1.

Surgical specialty treated as categorical, 7 categories: “general surgery” = reference category.

Method: Logistic regression, backward stepwise (PIN = 0.05, POUT = 0.10).

Model parameters: Valid cases = 457, Model Chi^2^ = 47.0, df = 8, p < 0.001. Nagelkerke’s R^2^ = 0.13.

The analysis of the relationship between anxiety and patient satisfaction showed a clear difference in total satisfaction (mean problem score out of 29 satisfaction questions) between patients who had anxiety (valid N = 141, mean total problem score = 22%) and those who did not (valid N = 333, mean value = 15%, p < 0.001, eta = 0.21) (Table 6).

**Table 6:** Bivariate analysis of problem scores (ANOVA).

		Valid answers	Mean problem score	Significance (ANOVA*)	Eta
Q1: Did you feel anxious regarding the upcoming anaesthesia before hospital admission?					
Total (29 items)	No	333 (70.3)	15.3 [12.1]	0.000	0.212
	Yes	141 (29.7)	23.3 [16.3]		
Dimension 1: Information and involvement in decision-making	No	331 (69.8)	13.6 [20.8]	0.000	0.187
	Yes	139 (29.3)	23.3 [28.5]		
Dimension 2: Respect and trust	No	333 (70.3)	1.94 [9.1]	0.000	0.162
	Yes	141 (29.7)	6.26 [17.2]		
Dimension 3: Delays	No	312 (65.8)	8.55 [23.6]	0.220	0.058
	Yes	132 (27.8)	11.7 [28.1]		
Dimension 4: Nursing Care in the recovery room	No	226 (47.7)	1.32 [9.1]	0.058	0.097
	Yes	119 (25.1)	3.78 [16.2]		
Dimension 5: Continuity of care	No	330 (69.6)	49.5 [31.7]	0.015	0.112
	Yes	141 (29.7)	56.8 [24.7]		
Dimension 6: Pain management	No	211 (44.5)	7.61 [23.2]	0.912	0.006
	Yes	98 (20.7)	7.31 [22.1]		

Values are absolute numbers, relative numbers (%), mean problem scores (%) and SD [xx].

Concerning the six sub-dimensions of quality of care, significant differences in the rating were also found for dimension 1 “information and involvement in decision-making” (14% of deficits stated in the non-anxious group compared to 23% in the anxious group, p < 0.001, eta = 0.18), dimension 2 “respect and trust” (2% vs 6%, p < 0.001, eta = 0.16) and dimension 5 “continuity of care” (49% vs 57%, p = 0.015, eta = 0.11). For dimensions 3 “delays”, 4 “nursing care in recovery room”, and 6 “pain management” there was no significant difference in satisfaction between the patient with and without anxiety (Table 6).

## Discussion

In this study, we assessed the prevalence and specific causes of perioperative patient-reported anaesthesia-related anxiety, a possible association between anxiety and overall patient satisfaction, and the sub-dimensions of this patient-reported outcome, measured with a psychometrically developed questionnaire. One-third of patients reported anaesthesia-related anxiety in the perioperative setting. Fear of not awakening from anaesthesia and PONV were the most reported causes of fear leading to perioperative anxiety in our study. We also observed a clear association between anxiety and impaired patient satisfaction. This was especially true regarding certain sub-dimensions of satisfaction: “information and involvement in decision-making”, “respect and trust” or “continuity of care.”

The prevalence of anxiety found in our study was in accordance with previous assessments using alternative questionnaires such as APAIS^15,16^, APAIS-A-T^4^ or STAI-scores^17,18^. Nonetheless, the range of reported prevalence of perioperative anxiety in the current literature is between 20 and 80%^19,20^. Many systematic reviews and meta-analyses did not differentiate surgery-related from direct anaesthesia-related anxiety, use different questionnaires, and use different time points within the perioperative pathway, resulting in this large range. Recent evidence supports our claim of separating these two types of anxiety, as the prevalence of anxiety related to surgery remains higher than anaesthesia-related anxiety^21^. However, further trials using validated tools and standardised settings to assess other factors influencing anxiety are required to better understand this range. In our study, women reported significantly higher anxiety levels than men. This result is supported by several previous studies with similar effect sizes in both inpatient and outpatient settings^16,22–26^. Age was negatively correlated with prevalence of anxiety, which is in accordance with current literature^15,27,28^.

Notably, we identified a different prevalence of anaesthesia-related anxiety depending on the surgical speciality. Current evidence assessing such differences is scarce but supports gynecologic procedures having the highest anxiety scores, as in our study^29^.

The specific causes of fear addressed in our study correspond to the findings of various studies on this topic^3,15,31^. The fear of not waking up was among the most reported fears, along with anxiety related to an error by the anaesthetist, fear of awareness, and fear of postoperative pain^3,15,19,23^. Anxiety triggered by the fear of developing PONV has not been frequently reported in the current literature assessing study populations with similar rates of general anaesthesia^15^. Regardless, as the risk of developing PONV is higher among women^32^, the patient population generally also reports higher anxiety. Because patients’ risk factors for PONV are investigated at the preoperative visit, they might be aware of their increased risk. Therefore, this result seems reasonable, and the lack of such data in previous studies might be caused by the use of questionnaires that are non-specific to anaesthesia-related anxiety and, thus, do not address PONV as a cause of fear.

Regarding the association of anxiety and overall patient satisfaction as well as the subdimensions of this patient-reported outcome, we found a clear association between patients with anxiety and impaired patient satisfaction. This was especially true regarding certain subdimensions of satisfaction: “information and involvement in decision-making”, “respect and trust” or “continuity of care”. Thus, even though over 60% of patients who reported anxiety in the perioperative period were able to talk about their concerns with an anaesthetist, the aggravating effects of existing anxiety on patient satisfaction indices could not be mitigated simply through having a contact point to talk with regarding the concerns. Therefore, other strategies are required to lower the rate of dissatisfaction with perioperative care among anxious patients.

It is worth considering that inherently less satisfied, and thus perhaps more critical, patients might experience more anxiety. However, it is the responsibility of anaesthesia care specialists to address this problem.

Current literature regarding “adequate” delivery of information^33,34^, the importance of continuity of care^11^, and respect and trust^35,36^ emphasize the importance of measures to improve those aspects for both better patient satisfaction and less anxiety as individual quality parameters of anaesthesia care. However, the association and possible implications of the two interrelated factors through a simple intervention have not yet been investigated.

Regarding the sub-dimension “continuity of care”, modern anaesthesia services are limited in the scope of actions mostly by the development of preoperative anaesthesia wards, thus divided care^7^. “Respect and trust” in the patient-anaesthetist relationship highly depend on the non-technical skills of the treating physician^37–40^, which have gained attention in resident and anaesthesia nursing training as well as continuing education only in recent years. Implementing timely changes to improve this dimension may not be possible through simple intervention^37^.

“Information and involvement in decision-making”, however, can easily be modified by adapting the timing, mode, and content of information delivery.

Timing did not affect perioperative anxiety in a systematic review including five trials assessing this factor^30^. The format of information delivery, for example using multimedia, verbal, or text information has been studied extensively^30,41–53^; however, these studies were conducted using various standards of questionnaires and study designs; therefore, a consensus is lacking.

Information content remains by far the least studied part of the dimension “information and decision-making”. Currently, the choice regarding the content of information provided to each patient is made by the anaesthetist and, to a certain extent, is provided in the law for safety and legal reasons^54–56^. The amount and depth of information given highly affects anxiety levels^57^. In addition, anaesthesia consultants remain the main source of information regarding anaesthesia as a basis for the patient-driven decision-making process.^58^ Our results indicate that the optimisation of preoperative information guided by patient-expectation^40,59^ is indeed a promising area of improvement for anaesthesia-related anxiety and patient satisfaction.

Still, our study has several limitations. First, it was conducted at a single medium-sized centre. Our results may not be generalisable to other settings, such as centres where mainly high-risk surgeries are conducted. However, the strict methodology, including unbiased sampling and high response rate, ensures that generalisability can be assured^60^. Second, the extent of anxiety was not assessed. This led to binary results (presence or absence of anxiety). The association between extent of anxiety and patient satisfaction should be assessed in subsequent trials. Third, the presence of a contact person does not give any information regarding the actual consultation by the patient. Therefore, it is not clear whether knowledge of the presence of someone is sufficient to ease anxiety or whether an actual interaction is needed. Also, patients with pre-existent psychiatric disorders were excluded in the development of the questionnaire. A confounding effect of mental disease on both anxiety and patient satisfaction as well as the actual consultation of a contact person need to be further assessed in subsequent trials. Finally, the questionnaire used in this study needs further validation for its use in identifying causes of fear and as an efficient screening tool to identify patients at risk for increased anxiety, in parallel to assessing patient satisfaction. Our approach of combining questions regarding perioperative anxiety and patient satisfaction in a questionnaire delivered after discharge may be impaired by recall bias. Nonetheless, the questionnaire was developed with a psychologist-orchestrated patient focus group (patients who underwent anaesthesia for elective surgery within 3 months and a minimum of one week after discharge before involvement in the development of the questionnaire) and included expert inputs from anaesthesia providers (nurses and physicians), administrative staff, and published literature^1^. It was psychometrically validated, and the content and construct validity were tested, internally consistent, and revalidated several times. Therefore, its validity in addressing the most important causes of fear is highly probable. In addition, we found a similar prevalence of anxiety as that in other scores and showed that anxiety affects the quality of care. Our questionnaire explicitly evaluated the perioperative period as a whole, which represents a strength of our method. Moreover, we standardised the timing of handling the questionnaires, as well as the sequence of all processes (pre-anaesthesia visit, contact and inclusion into the study, post-anaesthesia questionnaire, and follow-up) to prevent further bias. The delivery of the questionnaire to the patients’ homes after discharge to minimise a “social desirability bias” is an additional strength of our study.

In conclusion, we found that over one-third of the patients reported anxiety regarding anaesthesia, mostly related to the fear of not awakening from anaesthesia or PONV. Additionally, anxious patients reported higher problem-ratings for important quality of care dimensions, especially regarding “information and involvement in decision-making”, “respect and trust” and “continuity of care”, affecting patient satisfaction. Further research assessing not only the general desire for information but also the preferred content and ideal mode of delivery, especially in patients reporting anaesthesia-related anxiety, is needed to develop a strategic approach towards individualised care in the anaesthesiologic setting.

## Data Availability

All data generated or analysed during this study are included in this published article (and its Supplementary Information files).
