# Author Correction: Patient-reported perioperative anaesthesia-related anxiety is associated with impaired patient satisfaction: a secondary analysis from a prospective observational study in Switzerland

**DOI:** 10.1038/s41598-023-45525-1

**Published:** 2023-11-01

**Authors:** Corina Bello, Matthias Nuebling, Kira-Lee Koster, Thomas Heidegger

**Affiliations:** 1 Department of Anaesthesiology Spital Grabs, Spitalregion Rheintal Werdenberg Sarganserland, Grabs, Switzerland; 2 Department of Anaesthesiology and Pain Medicine, Bern University Hospital, University of Bern, Bern, Switzerland; 3 Data and Consulting, GDB mbH, Denzlingen, Germany; 4 Department of Medical Oncology and Haematology, Cantonal Hospital St. Gallen, St. Gallen, Switzerland

Correction to: *Scientific Reports* 10.1038/s41598-023-43447-6, published online 28 September 2023

In the original version of this Article, Kira-Lee Koster was incorrectly affiliated with ‘Department of Anaesthesiology and Pain Medicine, Bern University Hospital, University of Bern, Bern, Switzerland’. The correct affiliation is:

Department of Medical Oncology and Haematology, Cantonal Hospital St. Gallen, St. Gallen, Switzerland

The original Article has been corrected.

